# Synthesis of Superhydrophobic Barium Hexaferrite Coatings with Low Magnetic Hardness

**DOI:** 10.3390/ma15217865

**Published:** 2022-11-07

**Authors:** Arsen E. Muslimov, Makhach Kh Gadzhiev, Vladimir M. Kanevsky

**Affiliations:** 1 Federal Scientific Research Centre “Crystallography and Photonics” of Russian Academy of Sciences, Shubnikov Institute of Crystallography, 119333 Moscow, Russia; 2 Joint Institute for High Temperatures, Russian Academy of Sciences, 125412 Moscow, Russia

**Keywords:** barium hexaferrite, sapphire, magnetron deposition, low-temperature plasma, magnetization, superhydrophobicity, coercive fields, roughness

## Abstract

Using the multifunctional material barium hexaferrite as an example, the prospects for treatment at a quasi-equilibrium low temperature in an open atmosphere to form superhydrophobic magnetic coatings with pronounced crystalline and magnetic anisotropy have been demonstrated for the first time. The relationship between plasma treatment conditions, structural-phase composition, morphology, and superhydrophobic properties of (0001) films of barium hexaferrite BaFe_12_O_19_ on C-sapphire is studied. X-ray photoelectron spectroscopy (XPS), X-ray diffractometry (XRD), scanning electron microscopy (SEM), atomic force microscopy (AFM), as well as magnetometry and moisture resistance analysis, were used as research methods. During plasma treatment with a mass-average temperature of 8–10 kK, intense evaporation and surface melting were observed, and texturing of the deposit along (0001) is found. When the treatment temperature was reduced to 4–5 kK, the evaporation of the material was minimized and magnetic and crystal anisotropy increased. However, the increase in the size of crystallites was accompanied by the transition of oxygen atoms from lattice nodes to interstitial positions. All samples exhibited low coercive fields below 500 Oe, associated with the frustration of the magnetic subsystem. Features of growth of materials with a wurtzite structure were used to form a superhydrophobic coating of barium hexaferrite. Plasma treatment regimes for obtaining self-cleaning coatings are proposed. The use of magnetically hard barium hexaferrite to radically change the properties of a coating is demonstrated herein as an example.

## 1. Introduction

Plasma treatment, due to its undeniable advantages, has been used for many years in the production of materials and coatings. This is facilitated by such unique associated merits as the possibility of controlling the depth of impact, the ability to process objects of any shape, the low cost of processing, environmental friendliness, and, most importantly, the ability to cover large areas. The main types of plasma include high-temperature, as well as low-temperature thermal and non-thermal (cold) plasmas [1]. High-temperature plasma achieves ultra-high outputs and temperatures up to 10^8^ K, and, accordingly, any contact with a solid body leads to its destruction. The most widely used today in the synthesis of new materials and coatings is cold plasma at a maximum temperature of up to 10^2^ K, which makes it possible to modify a surface, its porosity, chemistry, adsorption, and catalytic characteristics without significant thermal action [2,3]. An intermediate position is occupied by a low-temperature thermal quasi-equilibrium plasma at a temperature of up to 10^4^ K. In this case, the plasma can be used as both a universal coolant and reagent at the same time. A high temperature makes it possible to create a gas phase in a short period of time, and subsequent chemical transformations and condensation make it possible to synthesize nano and micropowders. Consequently, the use of low-temperature thermal quasi-equilibrium plasma is considered to be most optimal in the plasma–chemical synthesis of powders [4,5]. It appears that it is possible to control the degree of influence and structural-phase composition through the duration of plasma treatment, which will allow the control of the properties of coatings of various shapes and morphologies during the recrystallization of surface layers. In general, taking into account the possibility of controlling the shape of grains, their sizes, texture, and mechanical stresses during recrystallization, wide possibilities open up for modifying the morphology and properties of coating surfaces.

In the work herein presented, for the first time the possibility of modifying the morphology and properties of the surface of materials by short-term exposure to thermal low-temperature plasma is considered, using the example of M-type barium hexaferrite BaFe_12_O_19_(BaM).

Substituted M-type BaM is by far the most investigated magnetic material. The structure of M-type barium hexaferrite is similar to that of magnetoplumbite, with space group P63/mmc and is characterized by a strong saturation magnetization of 72 emu/g, a high Curie temperature of 450 °C, and a large crystal anisotropy of 17 kOe along the C axis [6]. A large value of coercive fields is associated with uniaxial anisotropy (theoretical value is larger than 7 kOe). BaM is a classical hard magnetic material with high permeability and is traditionally used in microwave devices [7], as well as permanent magnets in radio engineering, automation, instrumentation, and electronics. Recently, several novel promising directions for the use of BaM have been actively studied. In particular, the ability to absorb electromagnetic radiation makes it possible to create receiving antennas with selective radio absorption in the sub-terahertz range (0.09–0.1 THz) [8,9]. Magnetically induced ferroelectricity and giant magnetoelectric effects were discovered in BaM [10], which makes it a very promising high-temperature multiferroic. It seems to us that a new round of studies of BaM can be associated with its transition to a magnetically soft state. Soft magnetic materials are used today mainly as various magnetic circuits and also need to expand the range of applications. It is a rather challenging task to obtain a material such as BaM with a pronounced magnetic anisotropy, high saturation magnetization, and low coercive field. There are studies [11] wherein substitution is used to reduce the coercive fields in BaM, but this leads to a simultaneous decrease in the saturation magnetization. A more difficult problem is to preserve the magnetic anisotropy.

Besides, a significant disadvantage of any coating is low corrosion resistance. This can be improved by increasing its hydrophobic properties, especially under conditions of intense exposure to aggressive ions, in corrosive aqueous media, or when there is a high probability of freezing. With a huge variety of studies in the literature, there are no data related to the moisture-resistance (hydrophobic) properties of the BaM surface. Ferrites generally exhibit hydrophilic [12] properties, and only ferrite nanoparticles demonstrate weak hydrophobicity [13]. Methods for creating hydrophobic materials and coatings are presented in the most comprehensive review [14,15,16,17,18]. All methods are based on an integrated approach: surface texturing and coating with hydrophobic agents. Surface texturing is traditionally carried out by laser and chemical etching, plasma-electrolytic oxidation. Silane solutions, Teflon, and other moisture-repellent polymeric materials are used as hydrophobic agents. All texturing methods are laborious and often require the use of chemicals. In the case of the use of hydrophobic agents, the main problem is the strength of the adhesive contact and the temperature resistance of the coating, which determine the operational characteristics.

In our opinion, the most effective way to do it is using extreme external influence, e.g., plasma treatment, which is a process of high-temperature high-power exposure, accompanied by thermochemical reactions. Structures of the BaM type possess uniaxial anisotropy. Such structures are characterized by significant growth anisotropy [19]: under supercooling conditions, they form needles, rods, hexagonal plates, etc. It seems to us that, for materials like BaM crystalline, magnetic anisotropy, as well as a superhydrophobic state, can be achieved using short-term plasma treatment without hydrophobic coatings.

The paper studies the influence of the conditions of treatment with a low-temperature thermal plasma flow in an open atmosphere on the structural-phase composition, morphology, magnetic, and moisture-resistant properties of BaM film coatings on sapphire.

## 2. Materials and Methods

Sapphire plates with basic C (0001) orientation were polished by the chemical–mechanical method and used as substrates for BaM film. BaM was deposited at a temperature of 350 °C by the magnetron sputtering (AcademPribor, Moskov, Russia) of a BaFe_12_O_19_ ceramic target in an argon atmosphere (*р*_Ar_~5.6 · 10^−3^ Torr) to form an anion-deficient polycrystalline film (type I). As a source of nitrogen plasma, a DC plasmatron with vortex stabilization and an expanding channel of the output electrode was used. A plasmatron generates at its output a weakly diverging nitrogen plasma jet with a diameter of D = 8 ÷ 10 mm [20]. The parameters of the flow of high-enthalpy plasma were determined by spectral methods with the AvaSpec 2048 three-channel fiber-optic spectrometer operating at a spectral resolution of 0.2–0.5 nm. The spectrometer was used to monitor radiation (with a periodicity of 3–4 spectra per second) along the plasma flow axis in a spectral range of 240–1000 nm. The presence of a large number of NI atomic nitrogen lines in the spectra of nitrogen plasma allowed us to measure Т by the Boltzmann exponent method. In addition to safety, nitrogen plasma was used to dope the near-surface layer of BaM samples with a nitrogen impurity, which can enhance the moisture-resistant properties of the surface [21]. The samples were treated in an open atmosphere in two plasma regions with different average mass temperatures: 4 ÷ 5 kK (type M) and 8 ÷ 10 kK (type A). Treatment time is 30 s. A longer treatment led to the destruction of the sample.

For microscopic studies, the JCM-6000 (JEOL, Tokyo, Japan) desktop scanning electron microscope (SEM) equipped with equipped with an energy dispersive X-ray (EDX) microanalyzer and the Ntegra Prima (NT-MDT, Zelenograd, Russia) atomic force microscope (AFM) were used. The film thicknesses were estimated using the SEM images of the transverse sections of the samples. The roughness was calculated using the Nova software. X-ray studies were carried out on an X’PERTPRO diffractometer (PANalytical, Almelo, Netherlands) in the Bragg–Brentano “reflection” geometry using CuKα radiation (λ = 1.54 Å) with a Ni β-filter. For comparison, measurements using a commercial BaM powder were carried out, and the data from [22] were used.

The magnetic hysteresis curves were measured on a NUVO Mk2 vibrating magnetometer (Fairgrieve Molding Ltd., Washington, UK) in the magnetizing field range from −5000 to 5000 Oe in out-of-plane and in-plane geometries.

X-ray photoelectron spectroscopy (XPS) using the SPECS spectrometer (Specs, Berlin, Germany) equipped with Al and Mg anodes was used to determine the chemical composition of the surface layer of the samples. The spectrometer is metrologically verified. In this work, we used Mg anode excitation. The anode material was chosen in such a way that the useful signal did not overlap with the Auger lines. The spectra were recorded in the binding energy range from 0 to 1200 eV.

The hydrophobicity of sample surfaces was analyzed (contact angle ϑ was measured) using the sessile droplet method. The measurements were performed at a relative humidity of 40–45%. The optical visualization was performed using a digital photographic camera. A 5 mm^3^ water droplet was applied onto a substrate. The measurements were performed 30 s after the droplet was applied to achieve its stable state. The axis of camera objective was located at the level of the water-droplet–sample-surface interface. The contact angle was measured according to the procedure described in [23].

## 3. Results and Discussion

### 3.1. Study of Topography and the Elemental and Structural-Phase Compositions of BaM Coatings

**Figure 1:**
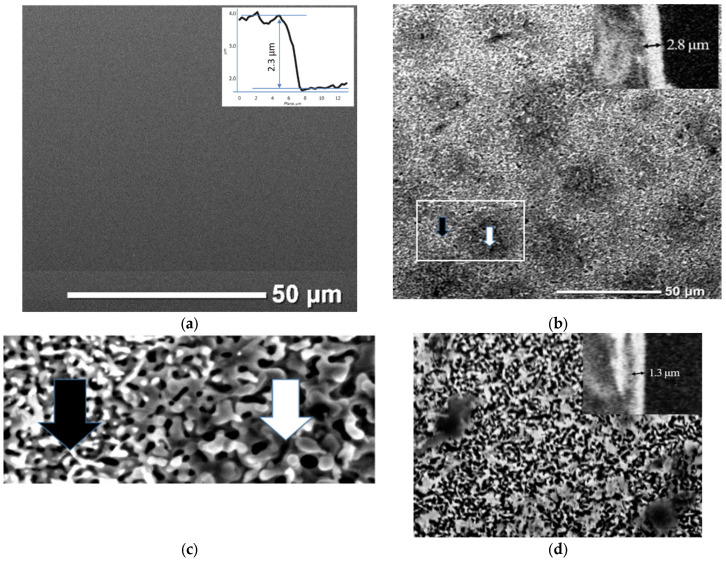
Microscopic images of BaM samples: type (I) (**a**), type M (**b**), type M, enlarged image of the regions indicated by dark and white arrows (**c**), type A (**d**).

**Figure 2:**
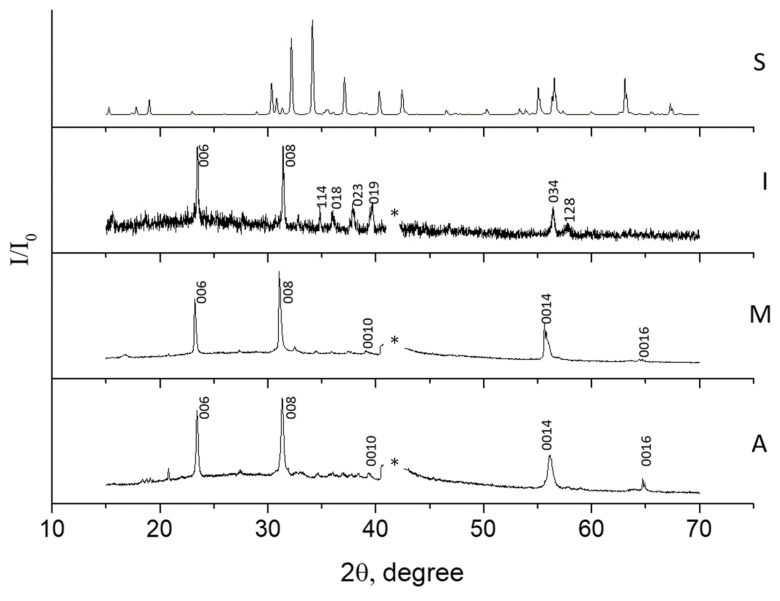
X-ray diffraction pattern of the corresponding BaM samples normalized to maximum. Insets: cross section images. Designations: *, reflection of the sapphire substrate. S—standart, I—initial, М—treatment at 4 ÷ 5 kK, A—treatment at 8 ÷ 10 kK.

At the first stage, a homogeneous type I film with a smooth surface (see Figure 1a), was formed using magnetron deposition in an argon atmosphere. According to the SEM data, the film thickness was 2.3 µm. The root-mean-square roughness (Rq) calculated from the AFM data was about 25 nm. According to X-ray diffraction (XRD) data, the initial type I BaM film was formed close to the polycrystalline (Figure 1b). After plasma treatment (in modes M and A), oriented BaM films were formed with a pronounced texture along the [0001] axis (Figure 2).

The reflections in the XRD pattern obtained for a commercial powder coincided with the results from [22] with a high degree of accuracy. Based on this, the interplanar spacing d along the [0001] texture axis of the synthesized films was calculated and compared with the data from [22]. In the case of types I, A, and M films, the values d_I_ = 0.2851 nm, d_А_ = 0.2855 nm, and d_М_ = 0.288 nm were obtained. This means that the crystal cell parameter of both films along the [0001] axis is slightly reduced compared with the data from [22] (d = 0.29 nm). The reason is the stoichiometry violation, lack of oxygen. It should be noted that there are no significant changes in the d_0001_ parameter during processing in the A mode. At the same time, processing in the M mode leads to an increase in the cell parameter and the approximation of the d_0001_ parameter to S standard data.

A comparison of the oxygen content in BaM samples on sapphire substrates is difficult since both the film and the substrate contain oxygen. According to EDX data, the content ratios of Ba/Fe atoms in type I, M, and A films are close, amounting to 0.110, 0.105, and 0.102, respectively. The ratio of the content of O/Fe atoms during heat treatment increased from 2.14 to 3.07; however, this was more of a qualitative result, which confirmed the increase in oxygen concentration. The nitrogen content was less than 1%. According to the SEM data, the film thicknesses at the cross-sections differed significantly: 2.8 µm and 1.3 µm for type M and A films, respectively. Thus, the decrease in the thickness of the type A film and the retention of the Ba/Fe ratio indicated congruent evaporation during the high-temperature treatment. The closeness of the parameters d_I_ = 0.2851 nm and d_А_ = 0.2855 nm confirmed the intense evaporation of the material during processing in mode A, while the diffusion activity of oxygen in the volume of the film did not have time to manifest itself. The diffusion coefficient in a wide temperature range was determined by the Arrhenius dependence equation (exponential). When processing in mode M, the diffusion activity of oxygen was at the maximum, which allowed it to penetrate into deeper layers. A higher temperature promoted crystallization.

**Figure 3:**
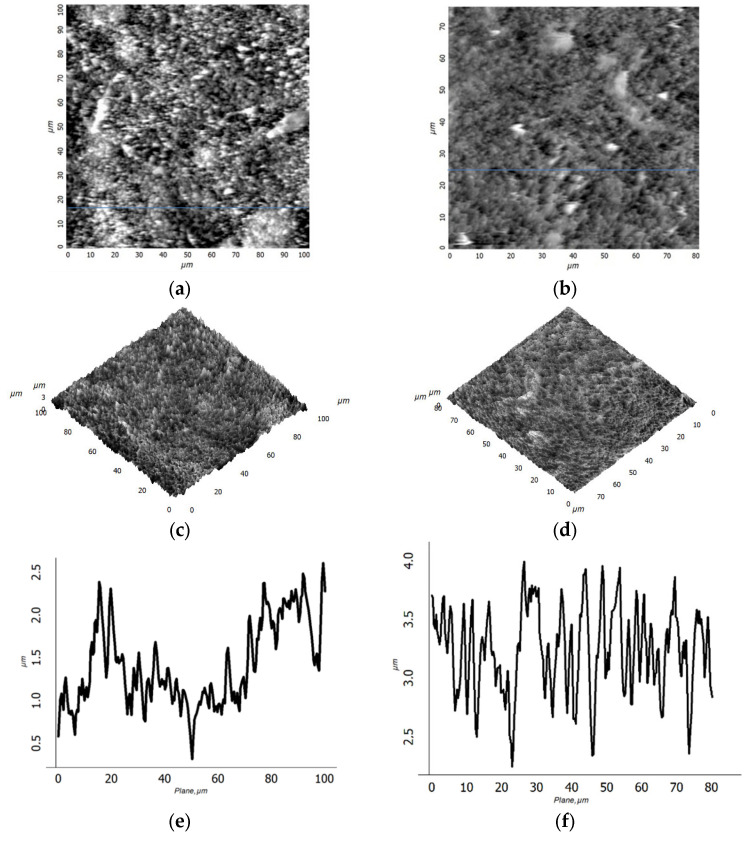
AFM images of 2D, 3D, and topographic cross-sections of the surface of type M (**a**,**c**,**e**) and type A (**b**,**d**,**f**) films, respectively.

Тype M films have a smaller FWHM of the {000n} family’s reflections, which indicates large crystallite sizes. A direct calculation of the coherent scattering region using the Scherrer formula gives an estimate of the crystallite sizes of 87 nm for type M film and 29 nm for type A film. In this case, the grains of type A film were larger and rounded according to the corresponding AFM image (Figure 1c). Taking into account the smaller size of crystallites of the type A film, it can be argued that large grains are formed as a result of sintering of individual crystallites. In general, there is likely to be a large temperature drop in the near-surface layers of the sample during the treatment in the A mode. The surface layers, which are in direct contact with the plasma, evaporate rapidly, but in the deeper layers, heating is insufficient for crystallization processes to occur. The film is partially melted from the surface and crystallized during quenching (Figure 1c). In the case of the samples treated in the M mode, the dependence of temperature on the depth of the layer was more uniform. The increase in the type M film’s thickness, in comparison with the initial type I film, was associated with its oxidation and an increase in porosity due to recrystallization after the melting stage during the treatment. According to the SEM (see Figure 3a–c) and AFM data, as a result of plasma treatment, the surface of the film was transformed from smooth to coarse-grained, with sharp relief drops up to several micrometers. Significant differences were observed in the roughness structure of the films (Figure 3).

For the type M film, bimodal roughness was observed (Figure 3e): against the background of the long-wavelength component of the geometric deviation from the surface plane, a short-wavelength component was observed. These data correspond to the SEM image (Figure 1a). The period of the long-wavelength part is about 50 µm, and the amplitude is about 0.5 µm. The presence of the long-wavelength part of the type M film’s surface roughness can be explained by the local melting and compaction of the material. Under conditions of a relatively low treatment temperature, as exposure to plasma increases, the temperature of the film rises, and, as a result, conditions for a phase transition (melting) arise. Melting is localized. Since the initiated phase transition in a certain region requires a constant heat supply, in addition to heat exchange with the plasma, heat flow from neighboring regions is observed. In turn, the temperature in neighboring areas decreases, and, as a result, the film surface acquires the shape of “honeycombs” (Figure 1b).

Comparisons of the root-mean-square (RMS) roughness according to AFM data for different scan sizes are carried out. For type M and A films, the following roughness values R_q_ were obtained: 338 nm (for M) and 238.8 nm (for A) in the case of a 10 × 10 µm^2^ scan; 349.9 nm (for M) and 588.9 nm (for A) in the case of a 100 × 100 µm^2^ scan. The scale dependence of the RMS roughness on the size of the AFM scan is known, and our results for type A films correspond to it. The presence of the dependence is evidence of the isotropic topography of the surface of the type A film. For type M films, the scale dependence is weak, which indicates a violation of surface isotropy.

**Figure 4:**
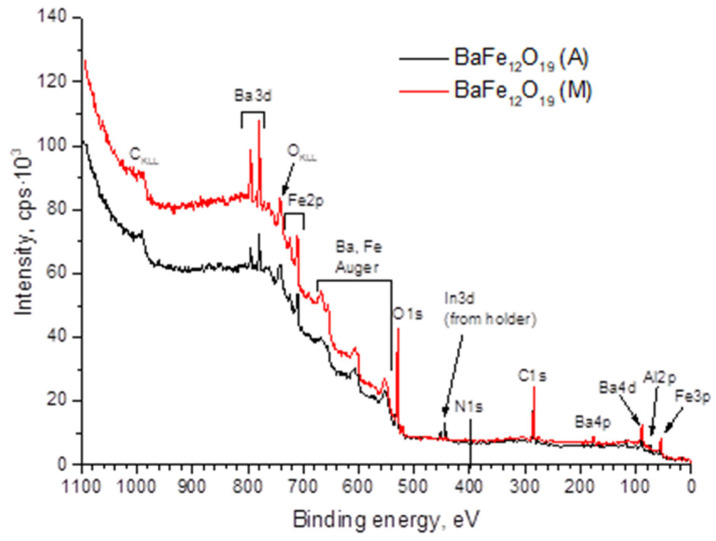
Panoramic XPS spectra of type M and A samples.

Hydrophobic properties are determined by the morphology and chemical composition of the layers in direct contact with the liquid. In this work, the XPS method was used to study the surface. All of the most intense lines of the elements that form the studied samples were observed on the panoramic XPS spectra of the samples (Figure 4). The presence of indium lines was associated with the substrate holder, while aluminum lines were associated with the presence of a sapphire substrate, on which the films under study were deposited. The presence of a large amount of carbon was associated primarily with the appearance of pollution.

The elemental composition of the near-surface layer calculated from XPS data is presented in Table 1. The increased barium content in comparison with the iron content in the near-surface layer can be seen, which does not correspond to the structural chemical formula of BaM. When processed the barium content varies depending on the processing mode. The high oxygen content may be due to adsorbed gases on the surface of the sample. The presence of less than 1% nitrogen can also be observed, consistent with EDX data.

**Figure 5:**
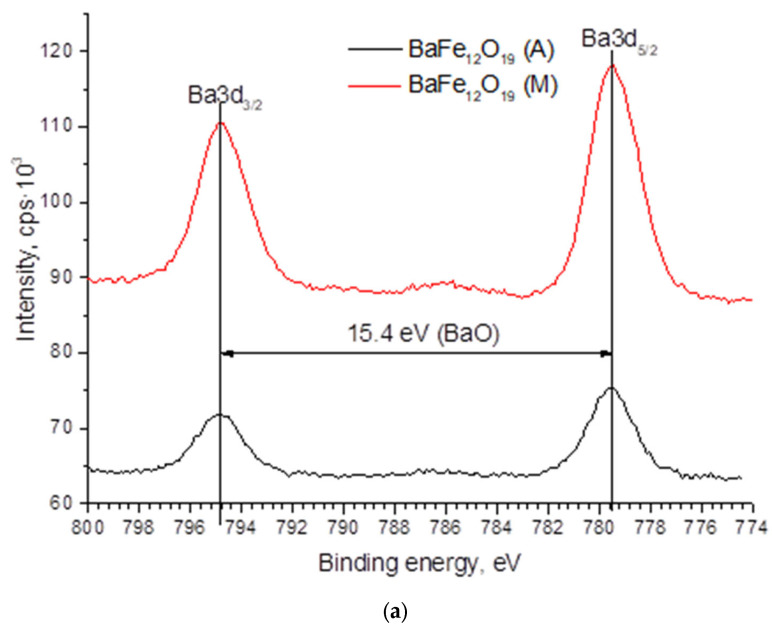

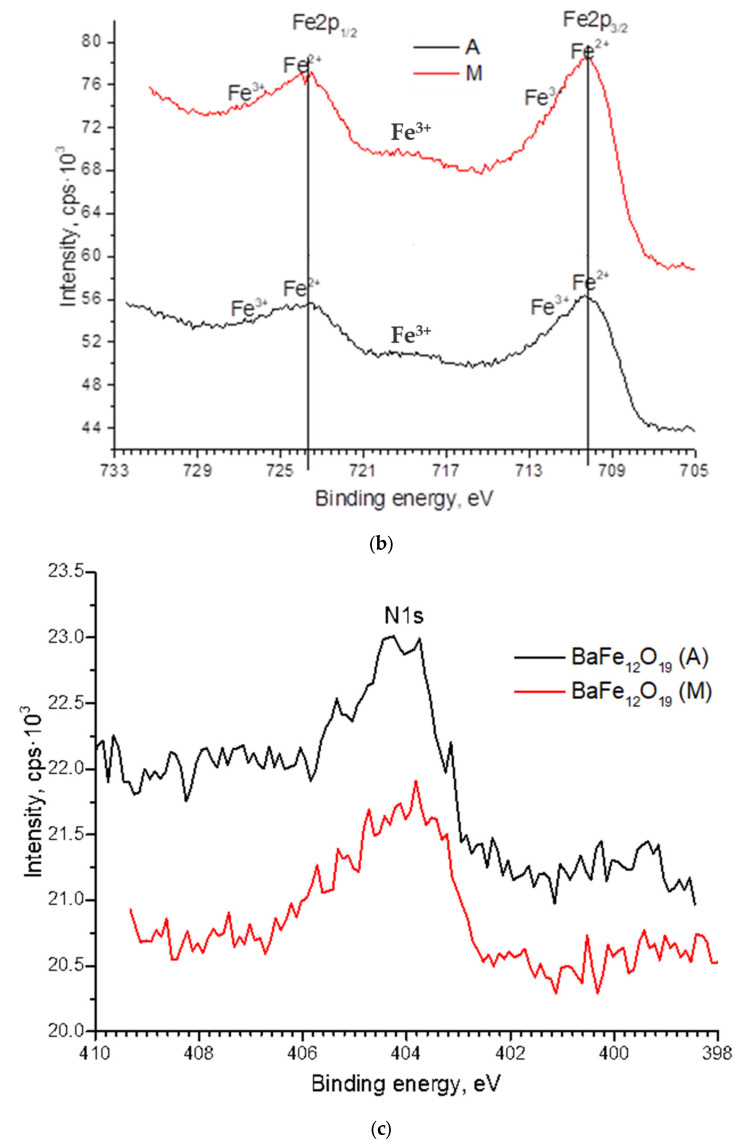

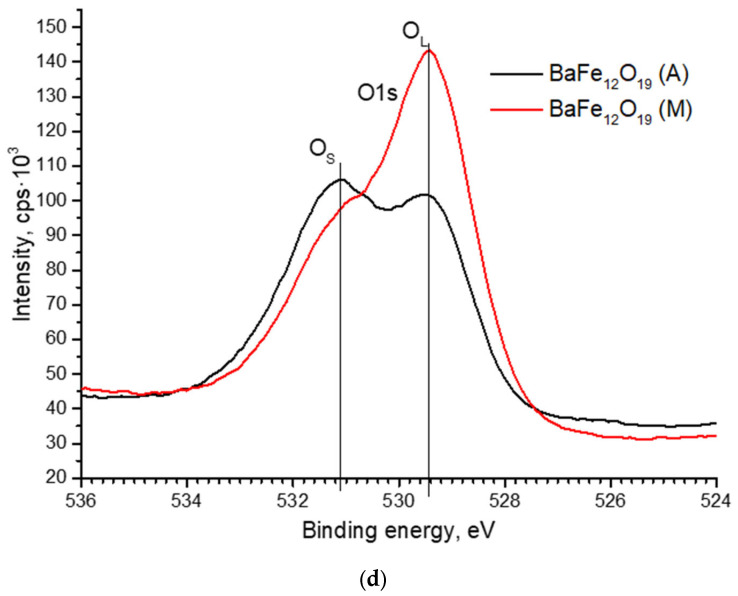
XPS spectra of Ba3d (**a**), Fe2p (**b**), N1s (**c**), and O1s (**d**).

In Figure 5 the spectra of Ba3d, Fe2p, O1s, and N1s are presented. The barium spectrum exhibits two peaks, Ba3d_3/2_ and Ba3d_5/2_, separated by 15.4 eV. This energy difference between the line maxima indicates the oxidized state of barium [24]. The magnetic properties of BaM are significantly affected by the valence state of Fe; therefore, the Fe2p_1/2_ and Fe2p_3/2_ peaks were studied in detail. The deconvolution of the Fe2p_1/2_ and Fe2p_3/2_ peaks indicates that the two main components are Fe^2+^ (724 eV), Fe^3+^ (726.6 eV) and Fe^2+^ (710 eV), Fe^3+^ (712 eV). A satellite peak at around 719 eV indicates the existence of Fe^3+^ [25]. It can be seen that the intensity of the 719 eV peak did not change during processing in different modes. The energy position and shape of the N1s nitrogen line (Figure 5c) most closely matches its state in relation to oxygen N-O [26]. Oxygen (Figure 5d) has two pronounced maxima O_L_ and O_S_, with energies of 529.5 eV and 531.1 eV, respectively. The O_L_ maximum is related to its bond in the lattice, and O_S_ is related to oxygen vacancies [27]. As indicated in Figure 5d, when processing in mode M, the intensity of O_L_ increases in comparison with mode A, while the intensity of Os decreases. This can be related to the transition of oxygen atoms from BaM lattice nodes to interstitial positions. Taking into account the increase in the thickness of the sample after treatment, it can be assumed that the oxygen atoms diffusing into the sample from the surrounding atmosphere also occupy the interstitial position. Thus, the approximation of the parameter d_М_ = 0.288 nm to the standard parameter d = 0.29 nm can be associated with lattice expansion due to interstitial oxygen atoms. For processing in high-temperature regime A, taking into account the closeness of the parameters d_А_ = 0.2855 nm to the parameter of the initial sample d_I_ = 0.2851 nm, as well as the XPS data, we can say that there were no significant changes in the precipitate: all elements were desorbed from the surface—therefore, a decrease in the concentration of individual components was observed (Table 1).

### 3.2. Magnetic and Hydrophobic Properties of BaM Coatings

**Figure 6:**
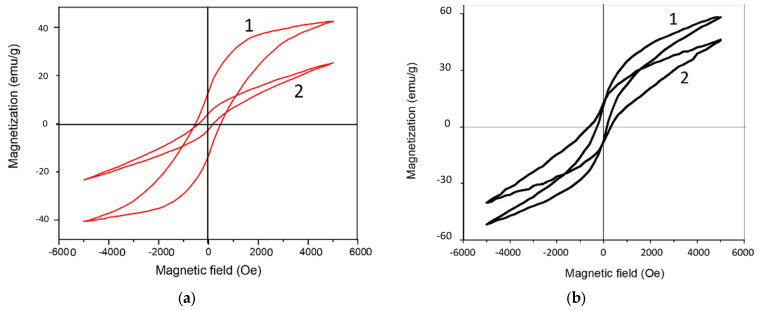
Curves of the magnetic hysteresis of BaM films processed in the M (**a**) and A (**b**) modes. Designations: 1—out-of-plane; 2—in-plane.

Figure 6 shows the magnetic hysteresis loops of BaM films. The results of the study of magnetic properties are presented in the Table 2 (M_s_—saturation magnetization, M_r_—remanent magnetization, M_r_/M_s_—rectangularity, H_c_—coercive field).

It can be seen that, in the case of M films obtained at a relatively low temperature, a pronounced magnetic anisotropy is observed. The dimensions of the [0001] crystallites in the M film are a maximum of 87 nm, which contributes to an increase in the magnetic anisotropy. In this case, the saturation magnetization is 42 emu/g. In A films obtained at high temperature, the magnetic anisotropy is weakly pronounced and the crystallite size is about 29 nm, but the saturation magnetization is higher and reaches 62 emu/g. It should be noted that the theoretical value of the saturation magnetization for BaM is 72 emu/g [28]. Generally speaking, many studies have been devoted to the dependence of saturation magnetization on the size of crystallites. In particular, the authors of [29] showed that saturation magnetization decreases linearly with an increase in the surface area of crystallites. The authors proposed a model of a crystallite containing a disordered noncollinear surface layer, the magnetic moment of which is not completely rotated, and an inner part, the magnetic moment of which can be aligned along the direction of the external field. An estimate of the domain size by analogy with [30] showed 1.8 µm for a type M film and 1.2 µm for a type A film. These results are in good agreement with the data of [31] and satisfy the relationship domene size σ ≈ T ^1/2^ for crystals with thickness 10 µm [32]. Accordingly, the magnetic domains in our BaM films are about 20–40 crystallites. With small amounts of crystallites constituting the domain, their inner part plays an important role, and the role of the surface is minimized. Despite the large size of crystallites in the type M sample associated with favorable crystallization conditions, oxygen is partially removed from the BaM lattice. Distortions in the crystal and magnetic structure as well as stresses are observed. An increase in the concentration of oxygen vacancies leads to a decrease in the number of Fe–O–Fe bonds, a weakening of exchange interactions, and the frustration of the magnetic system as a whole. Thus, when processing in the M mode, the magnetic anisotropy increases, due to the much larger size of the (0001)-oriented BaM crystallites. In this case, the saturation magnetization decreases due to the high concentration of point defects. For the same reasons, anion-deficient type A film demonstrates maximum saturation magnetization but less anisotropy.

The coercive fields for all samples are less than 500 Oe, which is more than 10 times less than the theoretical value of 6700 Oe [28]. Sample processing occurs under thermodynamically inhomogeneous conditions, when thermochemical reactions occur with a high rate of the propagation of the oxidation front from the surface deep into the sample. Under the conditions of the short-term plasma treatment of samples, the processes are closest to hardening, a feature of which is the fixation of mechanical stresses. The latter contribute to the preservation of the coercive field even with a significant decrease in magnetocrystalline anisotropy. The melting temperature of stoichiometric BaM is 1580 °C, and for anion-deficient samples is even lower. During high-temperature treatment, a molten phase is formed, which initially reduces the porosity of the sample and, during subsequent crystallization, forms regions of localized tensile stress. As is known [33], tension stresses increase saturation magnetization.

**Figure 7:**
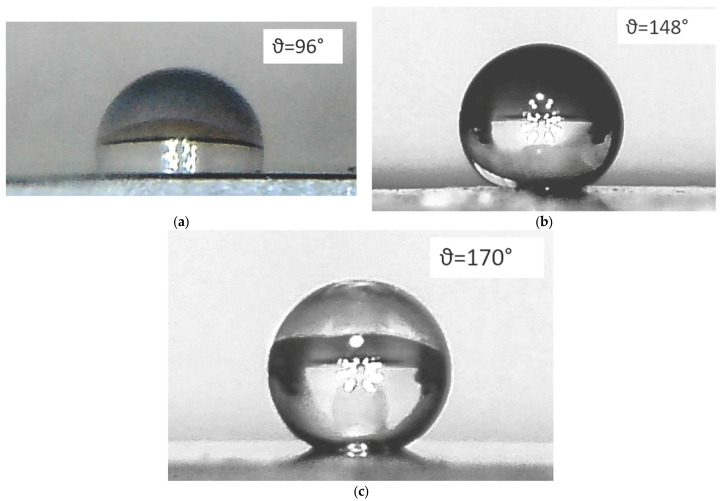
Optical images of the shape of a water drop on the surface of the samples: type I (**a**), M (**b**), A (**c**).

At the next stage, the hydrophobic properties (Figure 7) of the surface of the obtained samples were studied. The smooth type I film exhibited slightly hydrophobic properties (Figure 7a). The contact angle ϑ was 96°. Obviously, the hydrophobic properties of BaM are determined by the features of its crystal structure. The BaM hexagonal lattice consists of ten layers of large oxygen ions, four successive layers of which contain iron ions, and every fifth layer contains, along with three oxygen ions, a barium ion. Thus, in stoichiometric BaM, the moisture-resistant properties are determined mainly by the O–Fe bond in the surface layer. For hematite, such calculations are given in [34]. According to the XPS data (Table 1), the surface of both samples is enriched in barium, but its content decreases during treatment in mode A. On the other hand, an increase in the concentration of oxygen vacancies for a sample of type A should lead to an increase in surface energy and an increase in hydrophilicity. However, as the results show (Figure 7b,c), the surface exhibits hydrophobic properties. In our case, the main role in increasing hydrophobicity is played by surface texturing as a result of plasma treatment. Under conditions of heterogeneous wetting [35], an increase in roughness worsens the wetting of a hydrophobic surface. Studies (Figure 7b,c) and calculations showed that the contact wetting angles ϑ for the surface of samples of type M and A are 148° and 170°, and the proportion of the “liquid–solid” contact area is 0.19 and 0.03, respectively. The data obtained make it possible to characterize the M films as hydrophobic, and the A films as superhydrophobic. In order for the sample surface to be considered absolutely superhydrophobic, it is necessary to estimate the glide angles of a water drop, which should be on the order of 10°.

The studies carried out demonstrate the high water-repellent properties of the surface of a sample of type A. An inclination of the sample at an angle of 11° leads to the complete rolling of the water drops falling upon it. Superhydrophobicity is achieved due to the porosity and the specific structure of the film in the form of individual microcrystallites (Figure 7c), and the “lotus effect” is realized. In this regard, it is interesting to lower the contact angle (Figure 7b) for a sample of type M. In general, the difficulties of numerically estimating the roughness and size of surface relief irregularities present the main difficulty in analyzing the effect of surface topography on hydrophobic properties. In our study, the linear dimensions and shape of individual relief elements, according to AFM data (Figure 3a,b), are close. As the scale (scan size) increases, the visible differences in roughness increase. Calculations of the molecular dynamics of liquid droplets in contact with fractal surfaces are presented in [36]. According to these calculations, the contact wetting angle depends mainly on R_q_ and is almost independent of the fractal dimension of the surface. Thus, the superhydrophobicity of the type A sample can be associated with an increase in S_q_, which is most evident at large scan sizes. In addition, the long-wavelength component of roughness can play an important role. The presence of long-wavelength surface roughness with an average period of about 50 µm and amplitude of 0.5 µm can cause instability in a water drop on a macroscale: imbalance at the points of contact of the water drop with topographic “macroprotrusions”, the filling of “macrocavities”, and the redistribution of pressure inside the drop.

## 4. Conclusions

In the present work, the relationship between the plasma treatment conditions, structural-phase composition, morphology, and superhydrophobic properties of (0001) films of barium hexaferrite on C-sapphire is studied. Two treatment regimes were used: one with a mass-average plasma temperature of 4–5 kK and one of 8–10 kK. It is shown that the films obtained at a relatively low processing temperature have a pronounced magnetic anisotropy and a saturation magnetization of about 42 emu/g. Despite the observed magnetic anisotropy, the squareness of the magnetic curve is relatively low (~0.28). The films obtained at high temperature have a weak magnetic anisotropy but a higher magnetization value of about 62 emu/g. All films have low coercive fields below 500 Oe, which is more than 10 times lower than theoretical values. Plasma treatment leads to the texturing of the surface of the barium hexaferrite film coating, as a result of which it acquires superhydrophobic properties. To obtain self-cleaning coatings of magnetic barium hexaferrite with low coercive fields, treatment in plasma with a mass-average plasma temperature of 8 ÷ 10 kK is required. Using magnetically hard barium hexaferrite as an example, the possibility of using plasma treatment to radically change the properties of materials is demonstrated.

In the future, it is planned to investigate the effect of treatment with thermal low-temperature plasma on the ability to absorb electromagnetic radiation in the subterahertz range (0.09–0.1 THz), magnetically induced ferroelectricity, and magnetoelectric effects in BaM and also to extend the method of post-growth processing proposed in this work to improve the structure and properties of materials with pronounced uniaxial anisotropy, such as ZnO, GaN, etc.

## Figures and Tables

**Table 1:** Concentrations * (in %) of the main components of the films.

Sample	Ba	Fe	O	C	N
Type I	0.8	2.4	22.7	74.1	0
Type M	1.5	2.6	52.9	42.1	0.9
Type A	0.6	1.8	49.9	46.9	0.8

* Calculation was carried out for the spectra of Ba3d_5/2_, Fe2p_3/2_, O1s, N1s, and C1s.

**Table 2:** Magnetic properties of BaM films on sapphire.

		Out-of-Plane				In-Plane		
Sample	M_s_,emu/g	M_r_,emu/g	M_r_/M_s_	H_c_,Oe	M_s_,emu/g	M_r_,emu/g	M_r_/M_s_	H_c_,Oe
M	42	12	0.28	490	25	5	0.2	210
A	62	11	0.18	180	50	11	0.22	430

## Data Availability

Not applicable.
